# Investigating the Significance of Aerosols in Determining the Coronavirus Fatality Rate Among Three European Countries

**DOI:** 10.1007/s41748-020-00176-4

**Published:** 2020-09-20

**Authors:** Wenzhao Li, Rejoice Thomas, Hesham El-Askary, Thomas Piechota, Daniele Struppa, Khaled A. Abdel Ghaffar

**Affiliations:** 1 Schmid College of Science and Technology, Chapman University, 1 University Drive, Orange, CA 92866 USA; 2 Computational and Data Sciences Graduate Program, Schmid College of Science and Technology, Chapman University, Orange, CA 92866 USA; 3 Center of Excellence in Earth Systems Modeling and Observations, Chapman University, Orange, CA 92866 USA; 4 Department of Environmental Sciences, Faculty of Science, Alexandria University, Moharem Bek, Alexandria, 21522 Egypt; 5 Ministry of Higher Education and Scientific Research, Cairo, Egypt

**Keywords:** COVID-19, Coronavirus, Population density, Aerosol optical depth (AOD), Nitrogen dioxide (NO_2_), Sentinel-5P

## Abstract

The coronavirus pandemic has not only gripped the scientific community in the search for a vaccine or a cure but also in attempts using statistics and association analysis—to identify environmental factors that increase its potency. A study by Ogen (Sci Total Environ 726:138605, 2020a) explored the possible correlation between coronavirus fatality and high nitrogen dioxide exposure in four European countries—France, Germany, Italy and Spain. Meanwhile, another study showed the importance of nitrogen dioxide along with population density in determining the coronavirus pandemic rate in England. In this follow-up study, Aerosol Optical Depth (AOD) was introduced in conjunction with other variables like nitrogen dioxide and population density for further analysis in fifty-four administrative regions of Germany, Italy and Spain. The AOD values were extracted from the Moderate Resolution Imaging Spectroradiometer (MODIS) onboard the Terra and Aqua satellites while the nitrogen dioxide data were extracted from TROPOMI (TROPOspheric Monitoring Instrument) sensor onboard the Sentinel-5 Precursor satellite. Regression models, as well as multiple statistical tests were used to evaluate the predictive skill and significance of each variable to the fatality rate. The study was conducted for two periods: (1) pre-exposure period (Dec 1, 2019–Feb 29, 2020); (2) complete exposure period (Dec 1, 2019–Jul 1, 2020). Some of the results pointed towards AOD potentially being a factor in estimating the coronavirus fatality rate. The models performed better using the data collected during the complete exposure period, which showed higher AOD values contributed to an increased significance of AOD in the models. Meanwhile, some uncertainties of the analytical results could be attributed to data quality and the absence of other important factors that determine the coronavirus fatality rate.

**Electronic supplementary material:**

The online version of this article (10.1007/s41748-020-00176-4) contains supplementary material, which is available to authorized users.

## Introduction

In December 2019, many cases of pneumonia were reported in Wuhan, China. The subsequent investigation revealed the cause to be a novel coronavirus, which was later called 2019 novel coronavirus (SARS-Cov-2) or more commonly, the COVID-19 (Barcelo 2020). The virus that originated in China then spread across the world, becoming a global pandemic and affecting millions worldwide. According to Worldometers (https://www.worldometers.info/), the death count (as of Aug 24, 2020) due to the COVID-19 has surpassed 810,000, a number that probably underestimates the actual count, due to reporting issues (Lachmann et al. 2020; Cohen 2020; Bendix 2020). Though no vaccine is available yet (primarily due to the long time required for testing), studies continue in full flow (Lawton 2020; Mamedov et al. 2020). Apart from working to develop a vaccine, many researchers have investigated other factors that might impact coronavirus fatality. There are studies to evaluate the non-pharmaceutical interventions (NPI) such as social distancing, response of relevant agencies during the disease spread (Masrur et al. 2020; Li et al. 2020; Lai et al. 2020). Some of the other factors being studied include arctic oscillation (Sanchez-Lorenzo et al. 2020), air quality and wealth (Antonietti et al. 2020; Nichol et al. 2020), particulate matter pollution (Setti et al. 2020), diabetes (Means 2020), wind speeds (Coccia 2020), and ultraviolet radiation (Carleton et al. 2020), to name a few.

One of the environmental factors that received wide attention was nitrogen dioxide (NO_2_) reported by Ogen (2020a). That paper found that NO_2_ has a strong association with a high fatality rate of COVID-19 cases. The four countries selected in that study were France, Germany, Italy and Spain. However, Chudnovsky (2020) pointed out that Ogen’s study did not consider data of other factors that determine COVID-19 fatality and suggested that population density considerations would be important for a comprehensive analysis. Meanwhile, a response published by Ogen (2020b) agreed that the work did not prove any causality between NO_2,_ and the fatality caused by COVID-19; rather the study was meant to establish an idea and would need further exploration. An association analysis relating NO_2_ and population density to COVID-19 fatality has been done by at least one other study, by Travaglio et al. (2020). They found that both these variables are significantly related to COVID-19 fatality. Following up on these investigations, this study brings NO_2_, population density and aerosol optical depth (AOD) into the analysis, which attempts to see if adding AOD to the model, along with NO_2_ and population density, improves its predictive skill.

**Fig. 1:**
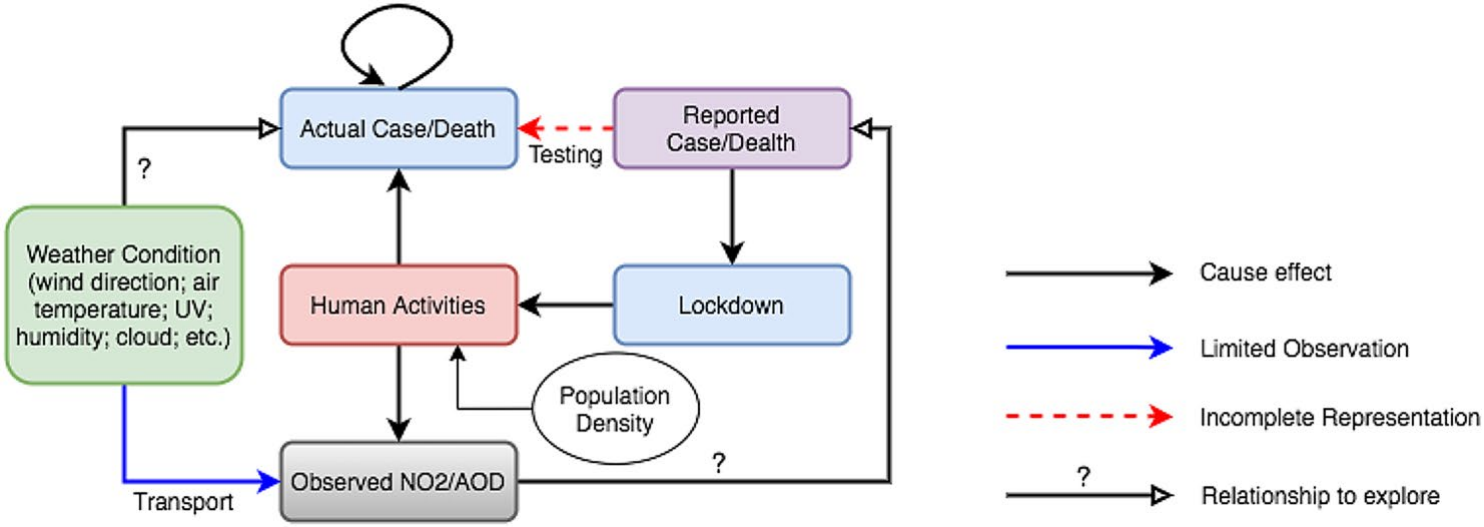
A diagram to show the environmental factors in the pandemic

Fifty-four administrative regions from three European countries (Germany, Italy and Spain) are investigated in this study. France was not included, since the total number of positive coronavirus cases per region in France was not available in time for our purposes; only the number of hospitalized cases per region was recorded. The European Environment Agency recorded around 76,200 premature deaths due to bad air quality in Italy, 73,900 in Germany and 33,300 in Spain for the year 2016 (EEA 2019). The population in these countries is thus exposed to air pollution which gives rise to adverse health impacts and getting worsened when infected by the coronavirus. In this paper, several tools such as AOD maps and regression models were used to explore the possible relationship between NO_2_, population densities, AOD, and the fatality rate of COVID-19 in the selected regions. This study is divided into two main parts—(1) pre-exposure period and (2) complete exposure period. During the pre-exposure period, NO_2_ and AOD were extracted during a time before the coronavirus fatalities were at their peak, that is, Dec 1, 2019–Feb 29, 2020. The hypothesis here is that pre-exposure may worsen the health conditions that are critical to the chances of survival after getting infected. During the complete exposure period, the NO_2_ and AOD data within the same time frame as the coronavirus fatalities were also included (Mar 1, 2020–Jul 1, 2020), along with the data from the pre-exposure period. This is based on the understanding that NO_2_ and AOD would be impacted during COVID-19 timelines due to human efforts such as lockdowns. Figure 1 is a diagram that displays some of the factors expected to impact the transmission of the virus, including weather conditions, human activities, and population density. There are several relationships open to be explored, such as the environment’s influence on the concentrations of NO_2_ and AOD, air pollutant’s relationship to the fatality rate, as well as human activities’ impact on the emissions of air pollutant. This study tests the hypothesis that aerosols are an important factor in coronavirus transmission rate (Asadi et al. 2020; Guo et al. 2020), which in turn would impact the coronavirus fatalities.

## Materials and Methods

### Data

#### The COVID-19 Fatality Data and Population Density Data

The COVID-19 fatality data were taken until Jul 1, 2020. For Italy, the COVID-19 fatality numbers per region were taken from the GitHub account (https://github.com/pcm-dpc/COVID-19) of the Department of Civil Protection, Italy. For Germany, data published by the Robert Koch Institute (https://www.rki.de/DE/Content/InfAZ/N/Neuartiges_Coronavirus/Situationsberichte/Gesamt.html) was used. For Spain, data maintained by the New York Times (https://www.nytimes.com/interactive/2020/world/europe/spain-coronavirus-cases.html) was used, who in turn retrieved it from the Spanish Ministry of Health. The death rate is calculated as the total number of coronavirus related deaths per region divided by the total number of coronavirus positive cases. For the regression models, the data for population density corresponding to each region was derived from the “citypopulation” program (https://www.citypopulation.de/). The “WorldPop Project Population Data” (www.worldpop.org) dataset retrieved by the Google Earth Engine platform (Gorelick et al. 2017) was used to show the population density distribution for the selected countries. The WorldPop dataset depicts the estimated number of people residing in each 1 km × 1 km grid cell.

#### NO_2_ Data

NO_2_ data were obtained from the onboard sensor, TROPOMI, on Sentinel-5 Precursor satellite version Near Real-Time (NRTI) (Veefkind et al. 2012). The data were collected and processed, from Dec 1, 2019 to Jul 1, 2020.

#### AOD data

The daily AOD data (Level 2, 1-km resolution) were obtained from MODIS Terra and Aqua using Multi-angle Implementation of Atmospheric Correction (MAIAC) (Lyapustin and Wang 2018). The green band (Optical_Depth_055) was used to extract the values and the cloudy pixels were masked for better quality. Like NO_2_, the data were collected and processed (e.g. filtered to remove the low-quality observations) from Dec 1, 2019 to Jul 1, 2020.

### Method

To assess the significance of AOD in predicting the COVID-19 fatality rate, a linear regression model was fit to the data, followed by use of the stepAIC() function from the MASS package (Venables and Ripley 2002) in R. The stepAIC() function performs stepwise model selection using AIC (Akaike information criterion) as the criterion for selection. Along with improving the model performance, this function helps to simplify a model through feature selection. This function also penalizes the model if more variables are added (Tripathi^2019^) and thus, is a good technique to determine whether an unrestricted model (having NO_2_, Population Density and AOD as independent variables) is better than a restricted model (having only NO_2_ and Population Density as independent variables). This helps to evaluate the significance of the third variable; AOD in this instance. The direction of the stepwise model selection is backward.

A beta regression model (Cribari-Neto and Zeileis 2009) was also fit to the data, given that the fatality rate lay in the standard unit interval of (0,1). The beta regression model provides good flexibility, which is delivered by the assumed beta law. The beta density assumes several different shapes that depend on the combination of parameter values, including left- and right-skewed, or the flat shape of uniform density. To assess the performance of the nested beta regression models (restricted model without AOD and unrestricted model including AOD), Wald test and Likelihood Ratio test were performed from the “lmtest” package (Zeileis and Hothorn 2002) in R. The Wald test statistic is given by,

$$W_{\text{T}} = I_{\text{n}} \left( {\hat{\theta }} \right) \left[ {\hat{\theta } - \theta_{0} } \right]^{2} ,$$

where $\hat{\theta }$ is the maximum likelihood estimator (MLE) and *I*_n_$(\hat{\theta })$ is the expected Fisher information (evaluated at the MLE) (Stephanie 2016a). The likelihood ratio test statistic is given by,

$${\text{LRT}} = \log_{\text{e}} \left( {\frac{{L_{\text{s}} \left( {\hat{\theta }} \right)}}{{L_{\text{g}} \left( {\hat{\theta }} \right)}}} \right),$$

where *L*_s_($\hat{\theta }$) and *L*_g_($\hat{\theta }$) are log-likelihood functions for the two models (Stephanie 2016b). These are statistical tests to evaluate the significance of variables and compare the goodness of fit between two nested (generalized) linear models.

## Results

### Distribution of Population in the Three Countries

**Fig. 2:**
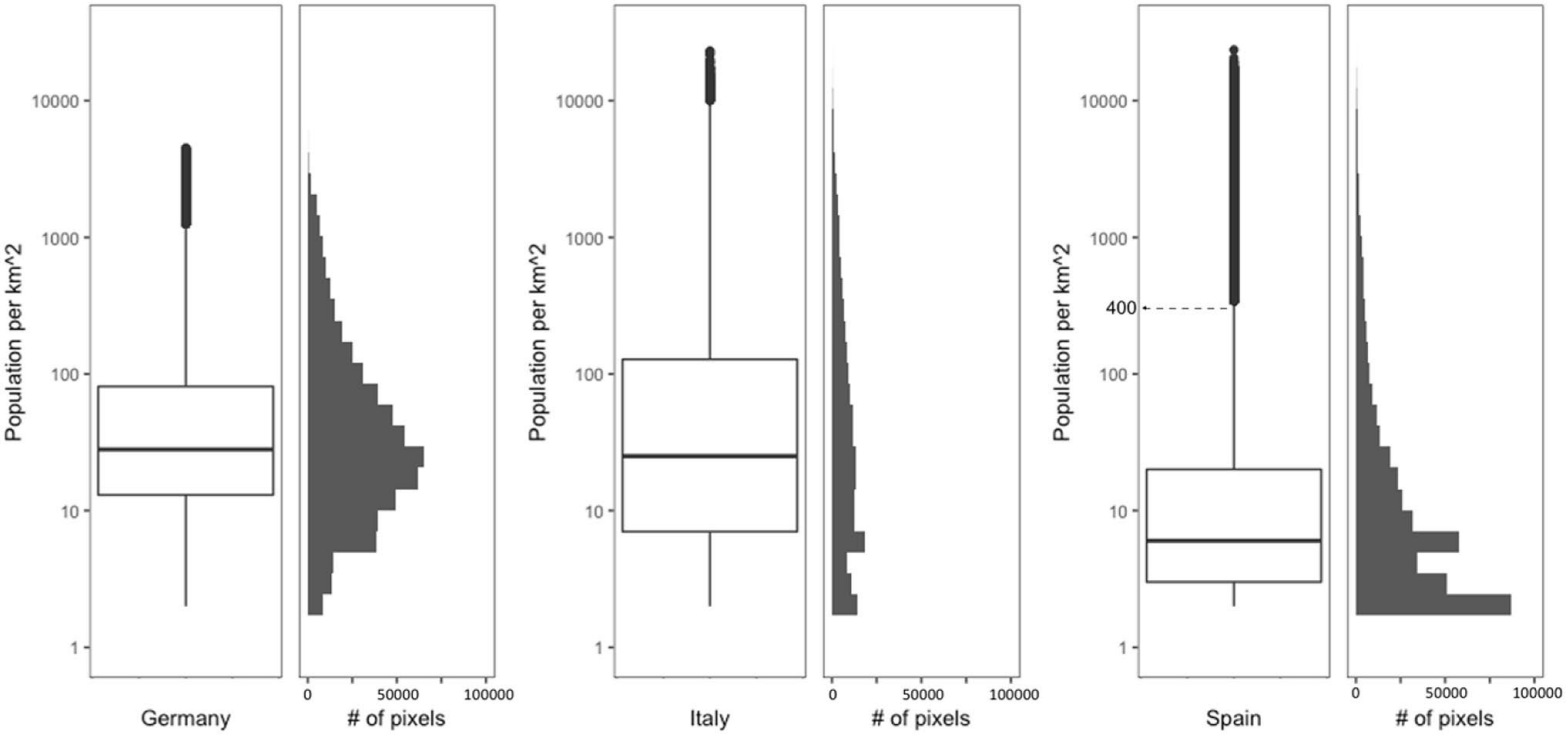
Boxplot and histograms depicting the population densities of Italy, Spain and Germany (pixels of population less than 1 person per km^2^ are not included)

The boxplots shown in Fig. 2 display the distributions of population density of Italy, Spain and Germany, with the y-axis showing the population per 1 km^2^ pixel. The histogram shares the same *y* axis with boxplot, with its *x* axis representing the number of pixels (equal to the areas in km^2^) having the same population values. Among these countries, Germany and Italy have similar median population densities (~ 30 people per 1 km^2^), which are much higher than Spain (< 10 people per 1 km^2^). Italy has a wider interquartile range (the IQR between 25th and 75th percentiles is shown between the upper and lower boundary of the box) than Germany and Spain have, indicating a more uniform distribution of population density in Italy. The cutoff outlier values defined for Germany, Italy and Spain are approximately 1000, 10,000 and 400 people per 1 km^2^, respectively. However, both Italy and Spain have a wider range of entire population density (from near 1 to over 10,000 people per 1 km^2^) than Germany. Therefore, it is concluded that: (1) Germany is mainly constituted with medium populated areas (10–1000 people per 1 km^2^), with limited areas of both densely (> 1000 people per 1 km^2^) and sparsely (near 1 person per 1 km^2^) populated areas, yet having no overcrowded area (> 10,000 people per 1 km^2^); (2) Italy has a more evenly distributed population, having both sparsely (near 1 person per 1 km^2^) and densely (> 1000 people per 1 km^2^) populated areas, as well as some overcrowded areas (> 10,000 people per 1 km^2^); (3) Spain has a skewed distribution with many areas having a limited population (near 1 person per 1 km^2^), while also having overcrowded areas (> 10,000 people per 1 km^2^).

### Spatial Distributions of AOD during Pre-Exposure Period

**Fig. 3:**
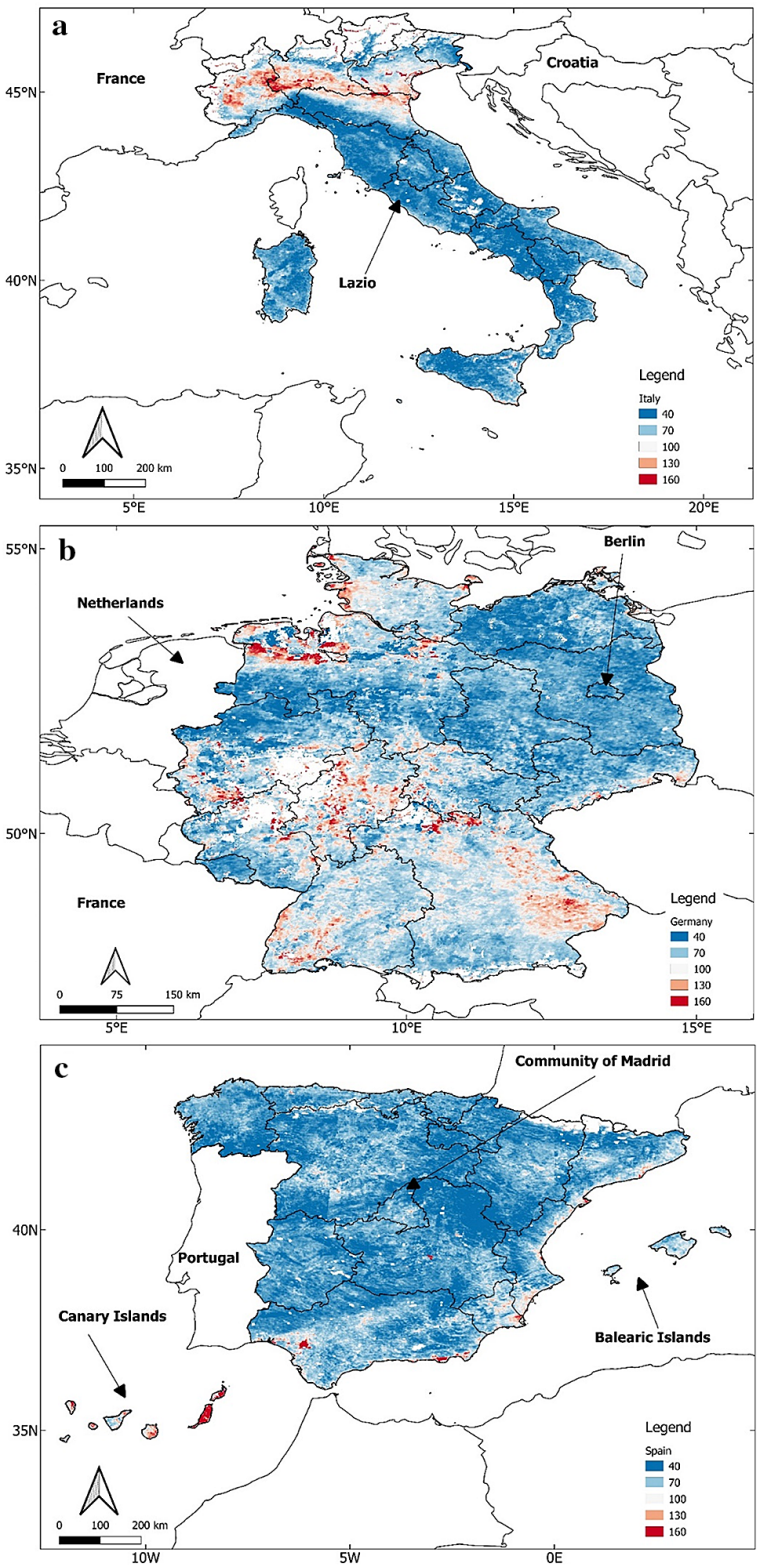
Spatial Distributions of AOD extracted from MODIS for all regions in a**a** Italy; **b** Germany; **c** Spain; for the pre-exposure period (Dec 1, 2019–Feb 29, 2020). The scale factor is 0.001. The actual AOD values displayed in the figure are in the range of 0.04–0.16. For Spain (**c**), the Canary Islands have been moved to fit in the figure

Figure 3 displays spatial distributions of AOD in the atmosphere for Italy, Germany and Spain. The time frame is from Dec 1, 2019 to Feb 29, 2020, which is the pre-exposure period.

By observing the maps alone, we can see Germany has higher values of AOD compared to Italy and Spain. Northern Italy, Southern Spain as well as the Canary and Balearic Islands of Spain all have relatively high AOD values.

### Regression Analysis During Pre-Exposure Period for NO_2_ and AOD

For the linear and beta regression models, data from 54 administrative regions of Italy (20 regions), Germany (16 regions) and Spain (18 regions) were used. Individual countries, and countries coupled with each other, were also evaluated to compare the results. For each combination, two models were generated: one, with NO_2_ and Population Density as independent variables, and another with NO_2_, Population Density, and AOD as independent variables. For a specific combination, a decrease in the AIC value or an increase in the adjusted *R*^2^ value determines the significance of the added variable (AOD). The stepAIC() function also helps in identifying the most important variables with the best (lowest) AIC value. Table 1 compares AIC, adjusted *R*^2^, and the variables which are considered most important by the stepAIC() function after the stepwise model selection.

**Table 1:** Results of linear regression models (pre-exposure)

	NO2_1 + PD			NO2_1^a^ + PD^b^ + AOD_1^c^		
	AIC	*R*^2^	Significant Variables	AIC	*R*^2^	Significant Variables
Italy	– 84.9894	0.3863	NO2_1	– 86.8784	0.4651	NO2_1, AOD_1
Germany	– 96.06988	0	Intercept	– 96.06988	0	Intercept
Spain	– 70.5783	0.3223	PD	– 72.2534	0.4106	PD, AOD_1
Italy + Germany	– 123.76349	0.0557	PD	– 123.76349	0.0557	PD
Italy + Spain	– 155.85485	0.2962	NO2_1, PD	– 155.85485	0.2962	NO2_1,PD
Spain + Germany	– 131.67629	0.4075	NO2_1	– 137.45427	0.5135	NO2_1, AOD_1
Italy + Germany + Spain	– 191.25573	0.1208	PD	– 191.25573	0.1208	PD

^a^NO2_1: NO_2_ from Dec 1, 2019–Feb 29, 2020

^b^PD: Population density

^c^AOD_1: AOD from Dec 1, 2020 to Feb 29, 2020

From the results, we can observe that AOD is a significant variable in 3 of the 7 models while in the others it is not. Some of the models have low adjusted *R*^2^ values, but for the models where AOD is found to be a significant predictor, the adjusted *R*^2^ values are relatively high (> 0.4). In those cases, there is an increase in the adjusted *R*^2^ value when compared to its restricted (NO_2_ + PD) counterpart. This increase in adjusted *R*^2^ value for the unrestricted model (NO_2_ + PD + AOD) suggests that the addition of AOD makes a significant contribution to that model. When Italy, Germany and Spain are included together (Italy + Germany + Spain), population density appears to be the most significant variable. The stepAIC() function dropped NO_2_ and AOD from the model as it did not consider them to be significant. For Germany alone, the models performed poorly, with neither of the 3 independent variables considered to be more significant than the intercept.

Table 2 compares the AIC values of the beta regression models by including and excluding AOD, along with NO_2_ and population density. It also shows the results of the Likelihood Ratio (LR) test and the Wald test performed on the two models to evaluate their performance. The significance level was set at 0.1. A *p* value of less than 0.1 indicates that the unrestricted model (including AOD) is statistically better than the restricted one.

**Table 2:** Results of beta regression models (pre-exposure)

	NO2_1 + PD	NO2_1 + PD + AOD_1	*p* value	*p* value
	AIC	AIC	LR test	LR test
Italy	– 82.06926	– 84.82103	0.0293	0.0143
Germany	– 94.25607	– 93.09458	0.3598	0.357
Spain	– 72.14794	– 72.93206	0.0952	0.1096
Italy + Germany	– 129.39762	– 127.54315	0.7028	0.6772
Italy + Spain	– 157.35442	– 155.44147	0.768	0.7754
Spain + Germany	– 141.70877	– 145.03185	0.021	0.0283
Italy + Germany + Spain	– 195.77259	– 193.96108	0.6642	0.6657

Again, the AOD appears to have some significance in three models. The AIC values improve, and the p-value suggests a significance too for Italy, Spain and Spain + Germany models. Similar tests were performed by excluding NO_2_ and population density. When investigating population density, 4 out of 7 model combinations had a *p* value less than 0.1 indicating its statistical significance while NO_2_ displayed significance in 3 out of 7 models, as did AOD. For pre-exposure, the results indicate a greater significance for population density in the COVID-19 fatality rate than when the significance of AOD and NO_2_ was investigated in these countries. The results of these investigations are attached in the Supplementary Materials section (Tables S1, S2, S3, S4).

### Spatial Distributions of AOD during Complete Exposure Period

**Fig. 4:**
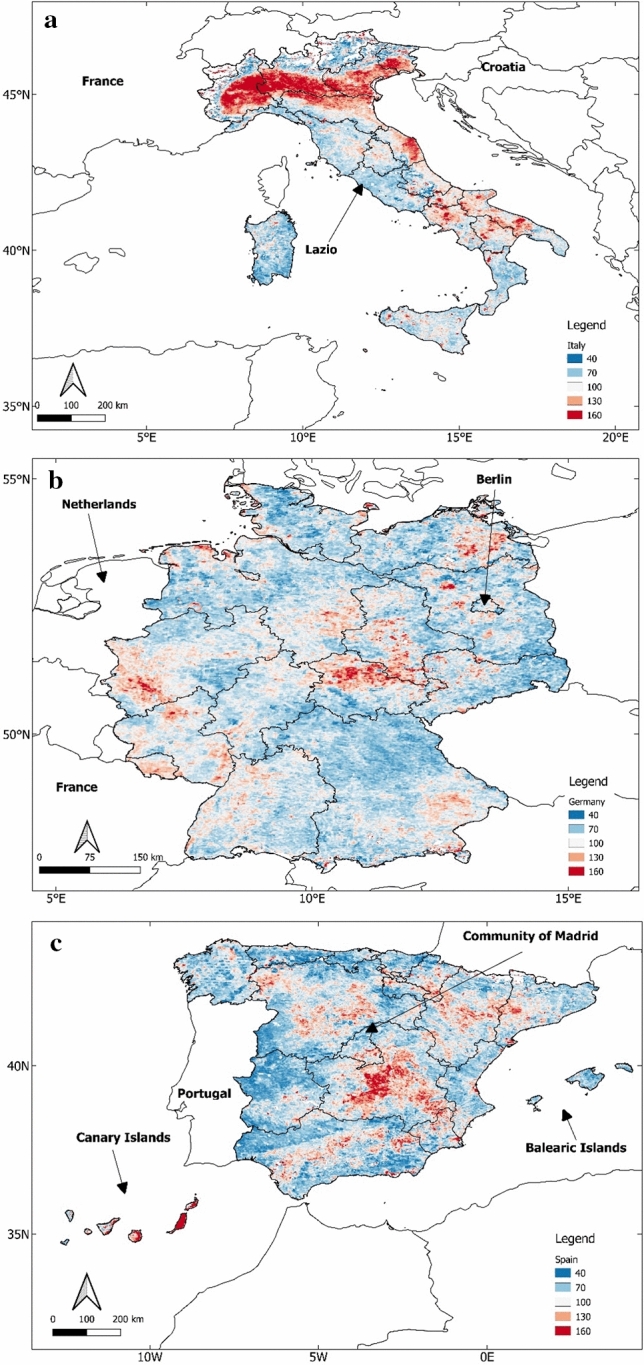
Spatial Distributions of AOD extracted from MODIS for all regions in **a** Italy; **b** Germany; **c** Spain; from Mar 1, 2020 to Jul 1, 2020. The scale factor is 0.001. The actual AOD values displayed in the figure are in the range of 0.04–0.16. For Spain (**c**), the Canary Islands have been moved to fit in the figure

Studies have shown that concentrations of both NO_2_ and AOD changed dramatically during the peak of the pandemic in countries like China and India. During this period, NO_2_ had a consistent decline across countries but AOD was impacted differently in different countries. China experienced an increase in AOD during the lockdown period while India recorded a decrease (Ghosh et al. 2020; Nichol et al. 2020; Ranjan et al. 2020). In view of this, NO_2_ and AOD data from Mar 1, 2020 to Jul 1, 2020 were also included in our analysis. This is the period when the coronavirus case-count had reached its peak. The AOD distributions during pre-exposure were displayed in Fig. 3. Figure 4 displays the spatial distributions of AOD in the atmosphere for the remainder period, which is Mar 1, 2020–Jul 1, 2020 in Italy, Germany and Spain.

There is a stark contrast between the AOD maps in Fig. 4 and the corresponding maps for the pre-exposure period (Fig. 3), especially over Italy and Spain, where the difference between the two periods appear to be larger. In Germany also there appears to be a slight increase in the AOD values from the pre-exposure period. This behavior appears similar to what was observed in China during the period when coronavirus cases were at their peak (Nichol et al. 2020).

### Regression Analysis During Complete Exposure for NO_2_ and AOD

NO_2_ and AOD data are divided into two periods and included in the regression analysis. Data from Mar 1, 2020 to Jul 1, 2020 (NO2_2, AOD_2) is also included along with data from the pre-exposure period Dec 1, 2019–Feb 29, 2020 (NO2_1, AOD_1). Table 3 displays the AIC, adjusted *R*^2^ values and the significant variables (from stepAIC()) for the linear regression models.

**Table 3:** Results of linear regression models (complete exposure)

	NO2_1 + NO2_2 + PD			NO2_1 + NO_2__2^a^ + PD + AOD_1 + AOD_2^b^		
	AIC	*R*^2^	Variables	AIC	*R*^2^	Variables
Italy	– 86.12189	0.4444	NO2_1, NO2_2	– 90.96363	0.5807	NO2_1, NO2_2, AOD_2
Germany	– 96.06988	0	Intercept	– 96.06988	0	Intercept
Spain	– 70.5783	0.3223	PD	– 71.72448	0.393	NO2_2, AOD_1
Italy + Germany	– 152.45341	0.5852	NO2_1, NO2_2	– 165.26975	0.7165	NO2_1, NO2_2, AOD_2
Italy + Spain	– 155.85485	0.2962	NO2_1, PD	– 155.85485	0.2962	NO2_1, PD
Spain + Germany	– 134.15914	0.4493	NO2_2	– 141.09301	0.5629	NO2_2, AOD_1
Italy + Germany + Spain	– 212.34928	0.4155	NO2_1, NO2_2	– 212.34928	0.4155	NO2_1, NO2_2

^a^NO2_2: NO_2_ from Mar 1, 2020 to Ju1 1, 2020

^b^AOD_2: AOD from Mar 1, 2020 to Jul 1, 2020

Multiple models selected AOD to be the significant variable and this is asserted in the increase of adjusted *R*^2^ values for those models. AOD showed its significance in 4 out of 7 model combinations. In Italy and Italy + Germany, the higher values of AOD during the current period (AOD_2) were considered significant while for Spain and Spain + Germany, the lower values of AOD in the previous period (AOD_1) was considered significant in determining the coronavirus fatality rate. Table 4 displays the AIC results from the beta regression models along with the *p* value from LR and the Wald tests for the same period as Table 3. The significance level was kept at 0.1. Thus, a *p* value less than 0.1 indicates that the unrestricted model (including AOD) performs statistically better than the restricted model.

**Table 4:** Results of beta regression models (complete exposure)

	NO_2__1 + NO2_2 + PD	NO_2__1 + NO2__2_ + PD + AOD_1 + AOD_2	*p* value	*p* value
	AIC	AIC	LR test	Wald test
Italy	− 83.32457	− 86.32696	0.0302	0.0125
Germany	− 92.96516	− 89.9271	0.6182	0.6251
Spain	− 70.18036	− 70.49765	0.1155	0.12
Italy + Germany	− 158.94446	− 167.82197	0.0016	0.0003
Italy + Spain	− 155.69081	− 151.73867	0.9764	0.9754
Spain + Germany	− 140.60018	− 143.37805	0.0337	0.0462
Italy + Germany + Spain	− 221.11899	− 218.81247	0.4288	0.4283

Complementing the linear regression results, some beta regression results also display stronger significance when AOD is added to a model. The *p* values from 4 out of 7 model combinations show significant contributions from AOD. Even the lower AIC values for those models suggest the same.

## Discussion

The regression models showed results regarding the significance of AOD in statistically explaining COVID-19 fatality rates, along with NO_2_ and population density. The statistical tests gave understandably mixed results in determining the significance of AOD, as environmental factors alone would not be expected to determine the fatality rate of a virus. The results from the complete exposure period (Dec 1, 2019–Jul 1, 2020) displayed a stronger significance of AOD than the results from pre-exposure (Dec 1, 2019–Feb 29, 2020). From the complete exposure period, 4 out of 7 models selected AOD as a significant variable with a subsequent increase in the adjusted *R*^2^ values too, indicating a better model when AOD was added as an independent variable to NO_2_ and population density. The AOD maps from the period (Mar 1, 2020–Jul 1, 2020) in Fig. 4 clearly show higher values of AOD than the maps from the pre-exposure period (Fig. 3). The results from the regression models during the complete exposure period are closely aligned with an article by Zhang et al. (2020) that discussed the indoor and outdoor airborne transmission of the coronavirus. Their study utilized space borne AOD measurements and was conducted for China, Italy and the United States of America (USA). It concluded that airborne transmissions do play a very important role in the spread of the virus. Though the AOD maps in Figs. 3 and 4 show that Germany has a decent prevalence of AOD, the models did not pick AOD as a significant variable there. In fact, the models did not pick NO_2_ and population density either when the regions in Germany alone were evaluated. It is known that these variables (NO_2_, Population Density and AOD) alone would not fully determine the fatality rate of the coronavirus. Other important factors work in synergy to increase the potency of the coronavirus. In some regions, the synergy might have been achieved due to these other factors, and thus led to the results showing the significance of AOD in models involving Italy and Spain. During the complete exposure analysis, the 4 models out of 7 selected AOD to be a significant variable. This would be the most important result from this study; namely, that when the AOD values increased, the models displayed an increased significance of AOD in determining the coronavirus fatality rate. The models were not a great fit, which could be due to a relatively small number of samples, the omission of other confounding factors, and some anomalies being missed using mean values (over 3 months) of NO_2_ and AOD per region. However, the models still gave valuable information regarding the significance of the variables. Adding data from other countries in Europe, along with the addition of other important factors could help in generating a better model. Also, looking at the higher AOD values contributing to better model performance, it is suggested that a similar study could be explored in regions with very high AOD values like the Middle East and North Africa (MENA).

## Conclusion

This study not only evaluated previous studies which showed that NO_2_ and population density are significant variables in determining COVID-19 fatality rates but also showed that AOD, used in conjunction with NO_2_ and population density, could help to improve the performance of the regression model to estimate COVID-19 fatality rate in regions with high AOD. The results from this study open the door for further scientific studies, using advanced modeling techniques and more accurate data, to determine different environmental characteristics affecting the COVID-19 fatality rate.

## Electronic supplementary material

Below is the link to the electronic supplementary material.

Supplementary material 1 (DOCX 16 kb)
