# Supplementary material for: Investigating the Significance of Aerosols in Determining the Coronavirus Fatality Rate Among Three European Countries
Source: Earth Syst Environ. 2020 Sep 20;4(3):513–22. doi: 10.1007/s41748-020-00176-4 (PMC7502156; doi:10.1007/s41748-020-00176-4)
Supplement: Supplementary file 1 — Supplementary material 1 (DOCX 16 kb) [file 41748_2020_176_MOESM1_ESM.docx]

**Supplementary Materials**

**Table S1.** Results of linear regression models investigating significance of PD

|  | **NO2_1+AOD_1** | | | **NO2_1+AOD_1+PD** | | |
| --- | --- | --- | --- | --- | --- | --- |
|  | **AIC** | **R^2^** | **Significant Variables** | **AIC** | **R^2^** | **Significant Variables** |
| Italy | -86.8784 | 0.4651 | NO2_1,  AOD_1 | -86.8784 | 0.4651 | NO2_1,  AOD_1 |
| Germany | -96.06988 | 0 | Intercept | -96.06988 | 0 | Intercept |
| Spain | -70.77646 | 0.3297 | AOD_1 | -72.2534 | 0.4106 | PD,AOD_1 |
| Italy+  Germany | -122.65839 | 0 | Intercept | -123.76349 | 0.0557 | PD |
| Italy+  Spain | -146.40299 | 0.0751 | NO2_1 | -155.85485 | 0.2962 | NO2_1,PD |
| Spain+  Germany | -137.45427 | 0.5135 | NO2_1,  AOD_1 | -137.45427 | 0.5135 | NO2_1,  AOD_1 |
| Italy+  Germany+Spain | -185.2757 | 0 | Intercept | -191.25573 | 0.1208 | PD |

**Table S2.** Results of beta regression models investigating significance of PD

|  | **NO2**_1**+AOD**_1 | **NO2**_1**+AOD**_1**+PD** | **p-value**  **LR test** | **p-value**  **Wald test** |
| --- | --- | --- | --- | --- |
|  | **AIC** | **AIC** |  |  |
| Italy | -84.94716 | -84.82103 | 0.2224 | 0.223 |
| Germany | -92.87112 | -93.09458 | 0.1182 | 0.1207 |
| Spain | -68.74761 | -72.93206 | 0.0136 | 0.0229 |
| Italy+Germany | -125.89078 | -127.54315 | 0.0528 | 0.0789 |
| Italy+Spain | -140.25853 | -155.44147 | 0 | 0.0013 |
| Spain+Germany | -146.5214 | -145.03185 | 0.4947 | 0.5355 |
| Italy+Germany+  Spain | -189.22893 | -193.96108 | 0.0096 | 0.0226 |

**Table S3.** Results of linear regression models investigating significance of NO_2_

|  | **PD+AOD_1** | | | | **PD+AOD**_1**+NO2_1** | | | |
| --- | --- | --- | --- | --- | --- | --- | --- | --- |
|  | **AIC** | **R^2^** | **Significant Variables** | **AIC** | | **R^2^** | **Significant Variables** |  |
| Italy | -85.19777 | 0.4182 | PD,AOD_1 | -86.8784 | | 0.4651 | NO2_1,  AOD_1 |  |
| Germany | -96.06988 | 0 | Intercept | -96.06988 | | 0 | Intercept |  |
| Spain | -72.2534 | 0.4106 | PD,AOD_1 | -72.2534 | | 0.4106 | PD,AOD_1 |  |
| Italy+  Germany | -123.76349 | 0.0557 | PD | -123.76349 | | 0.0557 | PD |  |
| Italy+  Spain | -149.1462 | 0.1395 | PD | -155.85485 | | 0.2962 | NO2_1,PD |  |
| Spain+  Germany | -121.15092 | 0.2141 | PD,AOD_1 | -137.45427 | | 0.5135 | NO2_1,  AOD_1 |  |
| Italy+  Germany+Spain | -191.25573 | 0.1208 | PD | -191.25573 | | 0.1208 | PD |  |

**Table S4.** Results of beta regression models investigating significance of NO_2_

|  | **PD+AOD**_1 | **PD+AOD_1+NO2_1** | **p-value**  **LR test** | **p-value**  **Wald test** |
| --- | --- | --- | --- | --- |
|  | **AIC** | **AIC** |  |  |
| Italy | -85.061 | -84.82103 | 0.1846 | 0.1805 |
| Germany | -94.61372 | -93.09458 | 0.488 | 0.4947 |
| Spain | -74.6926 | -72.93206 | 0.6246 | 0.6016 |
| Italy+Germany | -129.4599 | -127.54315 | 0.7729 | 0.7214 |
| Italy+Spain | -148.90054 | -155.44147 | 0.0035 | 0.001 |
| Spain+Germany | -129.32742 | -145.03185 | 0 | 0 |
| Italy+Germany  +Spain | -85.061 | -84.82103 | 0.452 | 0.3737 |
